# Post-Harvest Quality Evaluation of “Soreli” Kiwifruit at Two Ripening °Brix Values from Vineyards of Different Age Under Hail Nets

**DOI:** 10.3390/foods11030431

**Published:** 2022-02-01

**Authors:** Claudia Grasso, Roberto Forniti, Rinaldo Botondi

**Affiliations:** Department for Innovation in Biological, Agro-Food and Forest Systems, University of Tuscia, 01100 Viterbo, Italy; claudia.grasso@unitus.it (C.G.); forniti@unitus.it (R.F.)

**Keywords:** *Actinidia chinensis*, technological quality, phytochemicals, cover nets, post-harvest storage

## Abstract

The application of anti-hail nets is a practice that has been employed for a long time on different fruit and vegetable cultivations and in different fields of the world. In this work, we checked the effects of shading with white anti-hail nets on the post-harvest quality of “Soreli” kiwifruit collected at two different °Brix (7–8 and 8–9) from vineyards of two different ages (8 and 9 years) and stored at 1 °C for 90 days. It was observed that during the storage, the firmness and color parameters (L*, a*, b*, and Chroma) and the SSC content were generally higher in kiwifruit samples harvested in open field than in those under the nets. Regarding the bioactive compounds, the open field samples showed higher values in total flavonoids content during storage, and the content of carotenoids and chlorophylls in fruits grown in open fields was higher from 30–45 days up to the end of the storage. In contrast, the polyphenols and ascorbic acid values of fruits in open fields and under the nets showed a similar evolution of the values from 45 to 60 days. In general, the early and late harvest time based on the °Brix values and the different ages of the kiwifruit plants under the nets did not affect the quality parameters.

## 1. Introduction

Today, behind China, Italy is the second/third producer of kiwifruit of the world, together with New Zealand.

In Italy, the cultivation of kiwifruit began in the seventies and spread in the regions of the Centre-North, characterized by humid temperate climate. Kiwifruit vines are very sensitive to direct damage caused by hail in several countries [1], including in Italy [2]. Moreover, kiwifruit plants, due to their location, are more and more exposed to adverse weather conditions (wind, frost, and solar irradiation) as well as to attacks by insects (bugs), pathogenic bacteria, and moulds (*Pseudomonas syringae pv. Actinidiae* (PSA); *Phytophthora, Phytopythium,* etc.) [3,4,5]. Furthermore, in these last years, it is important to report also the onset of new diseases affecting the cultivation of kiwifruit, such as “kiwifruit vine decline syndrome, or KVDS”, usually associated with excessive precipitation/irrigation both in spring and in summer [5]. 

Since there are no specific actions to contain all these negative occurrences, all preventive actions must be implemented, including an active defense against rain, hail, and biological adversities with net canopies.

The use of nets represents one of the most effective systems to protect orchards and vineyards from these negative events. Nevertheless, due to the canopy cover material used, and its color and mesh size, some agronomic parameters of the plants and qualitative traits of the fruits can be negatively affected (decrease in vine fertility, fruit yield, and total soluble solids) [6,7]. Other studies on fruit tree crops displayed multiple responses to photo-selective shading in kiwifruits, apples, strawberries, table-grapes, and peach, including changes to photosynthesis rate, vegetative development, fruit-set, ripening rate, size, and quality parameters evolution [8,9,10].

The principal installation of nets in the Latium region (central Italy) to canopy kiwifruit vines is a solution that has been adopted by a number of growers for some years now. The variations of the physiological trends occurring on fruits and leaves are mainly due to the physical-chemical properties of the solar radiation (natural light, quality and quantity) reaching the tissue skin after filtering through the protective net [10,11,12,13]. Moreover, the kind of net (material type, color, section, etc.) also determines the thermal irradiation and humidity variations to which the plant tissues will be subjected [14,15].

The *Actinidia chinensis* “Soreli” is a yellow-fleshed cv. released in 2008 by the University of Udine (Italy). This cv. possesses many interesting agronomic and qualitative properties, such as early ripening compared to “Hayward” kiwifruit and a large/medium fruit size with excellent quality and nutritional trait [16,17], and it can be stored in commercial cold storage for a short period compared with the green-fleshed kiwifruit (between 80 and 120 days, depending on the season). In recent years, the chemical and nutritional, antioxidant properties of “Soreli” cv. during storage have been studied along with the management of the appropriate storage temperatures [18,19,20], but there has been no experimentation on this yellow kiwifruit coming from cultivations with net canopies.

In this work, we observed the evolution of the quality features of the fruits harvested at two different °Brix and stored for 90 days in cold rooms, from vines with different ages (8 and 9 years). In detail, we compared technological (firmness and color) and chemical parameters (SSC and TA) and bioactive compounds (carotenoids, chlorophylls, polyphenols, flavonoids, and ascorbic acid) of “Soreli” kiwifruit cv. obtained from outdoor cultivation and under-the-net canopies. The aim of this study was to evaluate the influence of vineyard shading nets on fruit quality during post-harvest storage.

## 2. Materials and Methods

### 2.1. Plant Material and Experimental Design

In the spring of 2018, white anti-hail nets (WH) were placed horizontally above the “Soreli” kiwifruit vineyards (20 ha) in the commercial orchards of “Tre Colli” farm located in Cisterna Campoleone (Velletri, Roma, Italy), and they remained in place for the duration of the experiment (summer 2020).

The nets were placed at 2 m above the tree, covering the entire vine rows, with no side protection, to allow even dispersion of the modified light at the canopy level, and good ventilation and gas exchange. The nets used in the cultivation of kiwifruit under experimentation were made of HDPE material (high-density polyethylene), and the color was pure white. The relative transmittance of PAR (photosynthetically active radiation) by the white nets was 81.2%, corresponding to 19.8 shading. The size of the nets was 7.8 × 4.0 mm, which allowed a barrier against hail and wind as well as an excellent balance between the penetration of light radiation and the control of heat fluctuation inside the orchard. 

Our study was conducted on four vineyards of “Soreli” cv. under nets and a control vineyard placed in open field (OF). Each of the experimental orchards measured ≈1 ha of soil surface and had ≈700 vines/ha, and the plants were spaced 4.0 m between rows and 3.0 m apart.

Ten vines from each of the five vineyards situated in diagonal orientation were selected on the basis of their position in the field and for a better availability of natural diurnal light exposure. From each of these vines, 100 fruits were harvested at two different maturity stages based on the SSC (7–8 and 8–9 °Brix), and the resulting fruits were compared to the vines grown in the OF (Table 1). 

The decision of these two ripening stages of the fruit (early and late plant harvest) of “Soreli” for the storage experimentation was related to a previous study reported by Cipriani et al. [17] who tested that early harvest (7.0 °Brix) improved overall quality compared to late harvest (8.5 °Brix) on the same yellow cultivar located in open field.

All the kiwifruit vines were grown on a soil with a clay texture and were managed using the same sustainable cultivation techniques. Irrigation was carried out through drop system in all the fields studied (about 4000/5000 m^3^ of water each field ha), and the same fertilizer treatments were used.

### 2.2. Storage Conditions

At harvest time, the fruits were selected for uniform size, appearance, and absence of defects and disease, packed in single-layer trays, and immediately transported to the Postharvest Laboratory of DIBAF (University of Tuscia, Viterbo, Italy). Kiwifruits were stored for 24 h at room temperature and were then gradually cooled from 15 ± 0.5 °C to 1 ± 0.5 °C for 12 days (3 °C every 2 days), to acclimatize to future low-temperature storage conditions (gradual cooling).

For three months, the cold rooms were maintained at 1 ± 0.5 °C with 85 ± 5% RH in normal atmosphere with potassium permanganate (KMnO₄) to absorb ethylene.

All analyses were performed at harvest time and after every 15 days of cold storage (0 days, 15 days, 30 days, 45 days, 60 days, 75 days, 90 days).

### 2.3. Chemical and Physical Parameters

Before storage, at each sampling time, and at the end of the experiment, the same 20 kiwifruits were weighed using a technical balance (Adam Equipment Co., Ltd., Milton Keynes, UK), to monitor weight loss during cold storage.

The percentage of weight loss (*WL*) was determined according to the following equation:

(1) $$\;WL=\left(\frac{W0-Wt}{W0}\right)\times 100\;$$

where *W*0 is the initial sample mass and *Wt* is the sample mass at time *t.*

At each sampling time, 20 fruits were analyzed to evaluate the total soluble solid content (SSC), the flesh color, and firmness. The fruits’ temperature was equilibrated to lab temperature before readings were done. 

The total soluble solid content (SCC) was measured on the fresh kiwifruit juice using a digital refractometer (ATAGO, Palette PR-32, Tokyo, Japan) and expressed as °Brix (%).

Flesh color was measured on peeled fruits using a Minolta colorimeter (Minolta C2500; Konica Minolta, Ramsey, NY, USA) to evaluate the chromaticity values L* (Lightness), a* (green to red), and b* (blue to yellow). The Chroma (C*) values were calculated as reported by McGuire, (1992) [21]. 

After removing a 1 mm thick disc of skin at the equatorial section of each fruit, the flesh firmness was evaluated destructively with a digital penetrometer (Mod. 53205; TR Turoni snc, Forlì, Italy) provided with an 8 mm tip, and the results were expressed as kilogram-force cm^−2^. The titratable acidity (TA) was measured in triplicate on the flesh juice obtained from five fruits. Each sample was titrated with 0.1 mol L^−1^ NaOH to a pH endpoint of 8.2, as indicated by a color change (clear to pink) using phenolphthalein indicator solution at 1%. The milliliters (mL) of NaOH used were recorded, and the results were expressed as milligrams of citric acid per 100 g of fresh weight (FW).

### 2.4. Bioactive Compounds Content 

The chlorophyll *a* (Cl *a*), chlorophyll *b* (Cl *b*) and total carotenoid (TC) content were extracted using 1 g of flesh tissue from five combined fruit samples homogenized in 10 mL of 99% (*v/v*) methanol with Ultra-Turrax and incubated overnight at 4° C. The mixture was centrifuged at 13,000× g at 4 °C for 10 min, and the optical density of the supernatant was measured at 666, 653, and 470 nm, using pure methanol as a blank sample. Pigment concentrations were calculated following the equation of Lichtenthaler and Wellburn [22]: 

Ca (μg mL^−1^) = 15.65A666 − 7.34A653

Cb (μg mL^−1^) = 27.05A653 – 11.21A666

Cx + c (μg mL^−1^) = (1000 A470 – 2.86Ca − 129.2Cb)/245

Total polyphenols (TP) and flavonoids (TF) content of methanol crude extracts was determined with Lambda 25 UV–Vis spectrophotometer (Perkin Elmer Instruments Ltd., Seer Green, Beaconsfield, U.K), according to the Folin–Ciocalteu [23] and aluminum chloride colorimetric [23] methods, respectively.

The polyphenols assay was performed by adding the methanolic extract to the assay mixture containing the Folin’s reagent and Na_2_CO_3_ at 7.5%. The reaction was developed at room temperature in the dark for 2 h. The absorbance was read at 765 nm, and the results were expressed as milligrams of gallic acid equivalents (GAE) per 100 g of fresh weight (FW).

For the determination of flavonoids content, the methanolic extract was added to a cuvette, followed by NaNO_2_ at 5% and AlCl_3_ at 10%, which were added 5 min later. After 6 min, 1 mol L^−1^ NaOH and distilled water were added up to a final volume of 2 mL. The assay was incubated at room temperature for 15 min. The absorbance was determined at 510 nm, and the results are expressed as milligrams of catechin equivalent (CE) per 100 g FW. All the analyses were performed in triplicate.

Ascorbic acid (AA) content was assessed according to [24]. Kiwifruit pulp (1.25 g) was homogenized with Ultra-Turrax in 5 mL extraction buffer, containing metaphosphoric acid (16% *v/v*) and EDTA (0.18% *w/v*). The homogenate was centrifuged at 12000× g at 4 °C for 10 min. The supernatant solution was assayed using 3% *(v/v*) metaphosphoric acid and 1:5 *(v/v*) diluted Folin’s reagent, adding deionized water up to a final volume of 2 mL. After 15 min, the absorbance of the sample was measured at 760 nm using Lambda 25 UV–Vis spectrophotometer (Perkin Elmer Instruments Ltd., Seer Green, Beaconsfield, U.K), and the ascorbic acid content was expressed as milligram of AA per 100 g FW. All the analyses were performed in triplicate.

### 2.5. Statistical Analyses

All results are expressed as the means ± standard deviation (SD). Statistical significances between different maturity stages of the fruit of different orchards and storage time were analyzed by two-way analysis of variance (ANOVA), and Tukey’s test at 5% level was calculated to compare differences between means. Differences at *p* < 0.05 were considered significant and are indicated with different letters.

## 3. Results

### 3.1. Chemical and Physical Parameters

The soluble solid content (SSC) initially increased quickly up to 60 days in all samples, and afterwards, during the storage, the WH values were rather homogeneous in all samples (Table 2). Although the fruits under the WH nets did not show a consistent increase in the SSC content between the two harvesting periods, the samples harvested in OF showed higher °Brix values than did those under the WH nets throughout the storage time. No significant differences were found during storage in the SSC content of orchards under the WH nets and with different age. At the end of the trial, the SSC values of fruits harvested in OF were about 3–4 °Brix higher than those of fruits covered by white nets. 

The titratable acidity values had not changed during cold storage between the two harvesting periods and between the fruits harvested in open fields and under white nets (Table 2).

The firmness, measured by destructive method, decreased progressively for all fruits harvested in OF and under the WH nets during cold storage (Table 2). 

Although the firmness measurement showed that the fruits of the vines grown in open field had higher firmness values than did the fruits under the white nets when harvested, there was a rapid and substantial loss of firmness for all samples after only two week of storage. Overall, the different samples had a uniform trend in terms of softening during the storage.

Weight loss increased linearly during cold storage, and no statistically significant differences were observed for all treatments (data not shown). 

Regarding the color parameters, lightness (L*), Chroma*, and b* parameters (Table 3) were lowest in fruits under the white nets compared to fruits from control in open field for the entire period of storage. The WH samples collected at 7–8 °Brix and at 8–9 °Brix showed an irregular decreasing pattern of Chroma values up to 30–45 days. Thereafter, the results exhibited a more homogeneous trend (with a slight rise) up to the end of the trial. The fruits from the first harvest kept higher lightness values during storage compared with the fruits harvested later. 

The chromaticity coordinate a* (Table 3) tended to have negative values that remained almost constant throughout the storage time for all fruits harvested in OF and under the WH nets. Regarding the harvest time, the samples remained stable until the end of cold storage.

### 3.2. Bioactive Compounds

The total carotenoids (CAR) and chlorophyll a and b (Cl a, Cl b) content of flesh at harvest was highest in all samples and then decreases sharply after two weeks of storage. However, the photosynthetic pigments values were higher in the OF samples than in the fruits under the WH nets from 60 days until the end of cold storage (Table 4). The fruits harvested at 7–8 °Brix maintained chlorophyll a and b concentration for a longer period (until 45 days) compared to those harvested later. During the storage and up to 45 days, orchard 4 had similar carotenoid values as those of OF and orchards 2 and 3, whereas orchard 5 had significantly lower carotenoid content (Table 4). 

Fruit polyphenol concentration (POL) did not exhibit a homogenous trend (Table 5). The vines in OF showed higher polyphenol content compared to the vines under the WH nets for up to two months (60 days). Kiwifruits from orchards 2 and 3 had similar polyphenol levels at 45 days of cold storage, and then significant decrease in POL content was observed in orchard 2 and a decrease in orchard 3 (Table 5). On the contrary, the fruits at 7–8 °Brix in orchard 4 had a higher POL content compared to the fruits of orchard 5 that were harvested later (Table 5). 

Moreover, the flavonoid content (FLAV) was higher in fruit harvested in OF throughout the storage time. Comparing the two harvesting periods, only significant differences were detected at the end of storage in the fruits of orchard 2. Based on the different age of trees, it is interesting to note that flavonoid values at the harvest time were significantly higher in fruits from vines of 8 years than in fruits from vines of 9 years. During the following storage, the flavonoids content tended to be uniform. (Table 5). 

The fruits in OF contained more ascorbic acid content (AA), which decreased with increasing storage time (Table 5). Instead, the AA content remained constant in the fruits under WH nets during the entire period of experiment, without significant differences between the two harvesting periods (Table 5).

## 4. Discussion

### 4.1. Chemical and Physical Parameters

The use of permanent cover protection or tunnel for the defense against rain, hail and biological adversities and to modify light intensity and internal microclimate represents an increasing option in the Italian and Latium areas dedicated to kiwifruit cultivation, especially in these last years when climate changes are more widespread and more severe compared to the recent past. These climatic disorders contribute to the development of harmful effects of both a biological (induction of bacterial, fungal, and pests) and physical (wind, hail, rain) nature on kiwifruit trees and also negatively affect the quality of the pre-harvest and harvest fruit (yield, moisture content, mechanical damage, and overall, the quality parameters of fruit).

Based on the quality of light and the dispersion of solar radiation, some fruit parameters undergo significant changes, as has been indicated in several studies [11,25,26]. Other work highlighted the photo-selective effects of protective shade nets of different colors (red, yellow, green, black, gray, and white) placed above orchards and showed inhibition of the phenomenon of excess photosynthetic photon flux density (PPFD), mainly caused by high temperature stress and water deficit (vapor pressure deficit) of leaves. Similarly, UV radiation and temperatures were found lower as a result of net filtrations [11,12,27]. The effect of overhead shading in kiwifruit vines during the growing season had adverse effects on plant fruit quality. The shading of “Hayward” kiwifruit vines caused a marked reduction in fruit quality (significant reduction of firmness and lower soluble solids concentrations) compared to unshaded fruits and a 50% reduction of flower number [7], whereas the shading nets increased leaf photosynthesis efficiency and fruit yield and quality in “Allison” kiwifruit vines [28]. In a work on three Chinese kiwifruit cvs, “Fengyue” (*Actinidia chinensis*), “Cuiyu” (*A. chinensis*), and “Miliang-1” (*A. deliciosa*), it was observed that shading during the summer season (30% light penetration) with temperatures above 33 °C could have positive effects on some quality aspects (fruit external appearance and fruit storage) but excessive shading could also induce plant stress [29]. Regarding the temperature variation between cultivations in OF and those covered by white nets, no specific experimental evaluations were conducted in our work, but some other research showed minimal seasonal variation between ± 0.4 and ± 1.3 °C when using white-colored nets such as those featured in our trial [30,31].

In our experiment, fruit firmness data at harvest were around 7 kg cm^−2^ for the control samples (OF) and about 6 kg cm^−2^ for fruit harvested at 7–8 °Brix and around 5 kg cm^−2^ for the fruit harvested at 8–9 °Brix of the vines placed under hail nets.

The firmness parameter evaluated through destructive analyses showed values that significantly drop down rather rapidly. These values are lower than those observed by Cipriani et al. [17] in “Soreli” cv. obtained by destructive analysis, where the values examined in two different years decreased less dramatically during storage for up to four months. Samples harvested in OF were significantly more consistent over the duration of the trial than were those under white nets for the two types of analyses performed. Amaranto et al. [12], in ”Gala” and “Fuji” apple cvs, and Snelgar and Hopkirk [7], in “Hayward” kiwifruit protected by white hail nets, also verified shorter fruit firmness during storage. In contrast, Basile et al. [30], in the same green fruit, and Gullo et al. [14], in “Jintao” yellow cv., showed no differences between fruit samples under white nets and the samples located in the open field.

The SSC found in “Soreli” samples during storage had higher values in uncovered control than in fruits under WH nets, regardless of the two different harvests. At the end of the trial, the fruits harvested in OF reached a significantly high value of 17.32 °Brix whereas the samples under WH nets only reached an average 14 °Brix (Table 2). This was not observed in “Jintao” at harvest time decided according to the fruit dry matter content and the Hue angle color value, in which the control samples in open field showed lower °Brix values than did those under the white nets [14]. Results on the evolution of SSC in fruits of *Actinidia deliciosa* harvested in OF and under the nets and stored in cold rooms showed conflicting results: Snelgar and Hopkirk showed higher values in uncovered control [7], on the contrary, Basile et al. [30] showed higher SSC values in fruit under the white, grey, and red nets than in control vines. In our experiment, it is plausible that the data on the firmness and SSC can be explained by the fact that shading the trees might reduce structural cell wall, fruit density, and storage carbohydrates, leading to a lower flesh firmness, reduced starch, and soluble sugar content, as has been assumed by Amarante et al. [12], in “Galia” apples. Regarding the occasionally conflicting results on fruit firmness and SSC mentioned in different works, it has been reported that the several impacts of seasonal orchard operations, crop load, and seasonal climatic changes may also be relevant [32,33].

Concerning the acidity content (Table 2), there were no significant differences between the treatments and during storage time, since the data showed consistent and homogeneous values. This is independent of the age of the kiwifruit vines. Some authors showed no effect on acidity content between fruit grown under white nets, such as those used in our trial, compared with the open field control on the “Jintao” cv. at harvest time [14] or on the “Gala” and “Fuji” apples at harvest and during the storage time at 0 °C. In addition, our data showed a generally uniform trend in the total acidity content in all samples during the storage, as previously verified in “Soreli” cv. in our precedent work [16], but not by Cipriani et al. [17], on the same yellow cv., which exhibited a decreasing trend of the acidity content during the storage.

The color parameters measured in this study showed L values that tended to decrease with storage time in both OF and WH nets samples, indicating a trend leading to a dark color and therefore an increase in flesh translucency (Table 3). However, L values of control fruits were significantly higher than those of fruits under the white nets, suggesting a lower tissue shift during the storage. The latter trend was also observed for early harvested kiwifruit samples in comparison to later harvested fruits, regardless of plant age. The color parameter b* (blue – yellow) during the storage and the Chroma values at harvest time and during the storage from 45 days and until the end of the trial showed higher results in fruits harvested in OF than in those under the nets and, in this case, the trends of this parameter were stable throughout the storage time. The values of a* (green – red) parameter did not show significant changes in color trend between samples during the storage. The data found in “Soreli” kiwifruit flesh color during cold storage could be related to an increase in metabolic degradation of carotenoids and chlorophyll *a* and *b* occurring in fruits under the nets during the storage (Table 4), as also observed by Snelgar and Hopkirk [7] in “Hayward” fruits. The effects of shading observed on the color parameters L, b*, and Chroma were assumed to be due to a reduction in the cumulative daily radiation incident upon a vine. Moreover, Davison [34] observed that fruits of *Actinidia deliciosa* cv. under the net were less green and less flavorful compared to those exposed directly to the sun. In contrast, Basile et al. [8] observed in another study that at the end of the cold-storage, the “Hayward” cv. flesh luminosity under the net was significantly higher than that of the uncovered control and the a* and b* colorimetric coordinates were only slightly affected by the nets. In another study on “Jintao” cv., higher values of L and color parameter b* (yellowness) were observed in fruits placed under the protective net than in fruits in OF at the time of harvest [14].

### 4.2. Bioactive Compounds

Throughout the post-harvest period, kiwifruit, specifically “Soreli” cv., are subjected to a series of physiological and metabolic changes of a catabolic nature that predisposes the fruit to a progressive deterioration, and the management of the storage affects the storage time of kiwifruit [16]. “Soreli” cv. is characterized by a high content of nutritional components, including the antioxidant compounds of “non-enzymatic” origin [18]. These antioxidants interact with ROS at the level of the cell membrane, acting as “scavengers” of these latter compounds and preventing membrane lipid peroxidation that leads to rapid cell deterioration [35]. In this sense, the antioxidant compounds are molecules that protect cells from the phenomenon of oxidative stress and rapid senescence of tissues [36]. Nevertheless, it is important to point out that the accumulation of phytochemicals during the production of plants depends on many factors, such as light quantity and quality, type of varieties or cultivars, growing season, and metabolic factors.

As previously observed in our work on the qualitative properties of “Soreli” fruit [16], the carotenoids content responsible for the yellow coloration of the fruit flesh was higher than the chlorophylls content that is associated with the green flesh color of “Hayward” kiwifruit. Regardless of the two different °Brix at harvest, the values of total carotenoids and chlorophylls *a* and *b* (Table 4) tended to decrease in all fruit samples from the harvest time, and significantly higher values were kept in OF samples from 30–45 days up to the end of storage. Therefore, the effect of the shading net did not seem to have any particular impact up to 30 days for carotenoids and up to 45 days for chlorophylls. In the following storage time, the loss of content tended to be much more marked in the fruits under the nets. The effect of shading on the concentration of carotenoids and chlorophylls (as well as on nutritional features) of kiwifruits is still an object of study. The data currently existing are largely based on research carried out with some types of nets with different colors on Hayward kiwifruit vines [7,30,37]. The studies on yellow kiwifruit are still only preliminary [14], and nothing is known about the “Soreli” cultivar. Covering with nets on “Hayward” kiwifruit vines, irrespective of their colors, plays a role in the reduction of daily intercepted light intensity and therefore in the reduction of chlorophyll content and this content was more severe than in the fruits harvested in OF, exposed to full sun, as has been observed by Antognozzi et al. [37] and Davison [34]. In the same cv., Snelgar and Hopkirk [7] found that shading with nets (45% or 70% shade) did not affect chlorophyll *a* and *b* concentrations during the storage, whereas Basile et al. [30] observed a significant decrease in chlorophyll and carotenoid content at the end of the storage time in samples under the nets compared to uncovered control. The mentioned authors assumed that the influence of photo-selective nets on the color of kiwifruit flesh may be due to photo-morphogenic effects rather than to a different availability of light. Finally, in fruits of the freshly harvested ’Jintao’ yellow cv., no differences in chlorophyll and carotenoid content were shown between fruits under the nets and fruits in OF [14].

At the initial time of storage, the values of fruits in OF tended to exhibit a marked increase in polyphenol content up to the maximum value at 45 days compared to the samples under the nets and then decreased up to the end of the trial to the values comparable to fruits covered by nets (Table 5). 

The evolution of flavonoids follows a different trend: the content of these compounds generally tended to decrease during storage in all samples, with a less evident trend in uncovered control (Table 5). These data can be explained through the evidence shown in some studies, in which it was verified that the radiation emitted in the UV spectrum is a major constituent of light that affects the concentration of phenols and polyphenols in various plant tissues [38,39,40]. It has been observed that the concentration of polyphenols increases when plant tissues of fruits and vegetables are exposed to UV light, and it has also been verified that flavonoids are the compounds able to absorb UV radiation by acting as a protective feature against tissue damage [41]. In this sense, it is possible to suggest that “Soreli” kiwifruit exposed for a long time to full sunlight have been able to accumulate more polyphenols and flavonoids during the storage than have fruits kept under white shading nets that partially minimized the UV light radiation exposure. In “Hayward” kiwifruit at harvest time, it was observed that fruits under the white net showed lower polyphenol and antioxidant content than did fruits in OF. After storage at −0.4 °C for five months, the polyphenol content of the samples under WH nets increased to values comparable to those of uncovered control [30]. In contrast, data of phenolic and flavonoid content detected in “Jintao” kiwifruit at harvest were higher in samples under the white nets than in those in OF, showing contrasting results to those observed on “ Soreli” cv. [14].

The turnover of the antioxidants during the vegetative growth of the fruit, in this case the ascorbic acid, which is included among the main components in kiwifruit, depends on many factors such as temperature, quantity and quality of light, type of cultivar, growing season, and field treatments. The environmental conditions are essential in the activation of biotic and abiotic phenomena associated with the response to the stress condition that leads antioxidants compounds, such as polyphenols and ascorbic acid in kiwifruit, to bind with reactive oxygen species (ROS) induced by the stress event, generating a protective cell response of plants [42]. In our study, we observed that “Soreli” fruits showed higher values in OF than did those covered by nets. Ascorbic acid content was higher in the presence of a high light intensity during the production period of fresh produce [43]. During storage, the AA content of the uncovered control was consistent up to 45 days and then decreased significantly. It is interesting to notice that samples under the nets had lower AA values at harvest and then maintained a homogeneous content comparable to that of control samples until the end of storage. Regardless of the different °Brix at harvest time and of the age of trees, the trend of AA and polyphenols content in “Soreli” kiwifruit under white net may indicate a possible mechanism of action on “Soreli” fruits. At harvest time, the intensity and quality of the lights seem to determine higher values of antioxidants, such as polyphenols and AA, in fruits harvested in OF, whereas in the following storage period, the fruits under the white nets showed constant values that suggest a possible “managed” metabolic response to stress, comparing to the control samples. This response to stress on fruits under the nets could be induced by the physical protection and the shading provided by the same nets (temperature, humidity, wind, chill, biological strikes) throughout the growing season, rather than being a direct effect of the qualitative nature of light on the nets. These observations are also confirmed by the data of antioxidant activity and polyphenols content provided by Basile et al. [30] in “Hayward” cv. at harvest time and after five months of cold storage. Bergquist reported in baby spinach that ascorbic acid concentrations were significantly lower in plants grown under the net than in unshaded plants [44]. On the contrary, Makus and Lester observed in mustard green that leaf ascorbate was higher in plants grown in ambient light than in plants grown in reduced light [45]. More recently, in “Jintao” kiwifruit cv., no differences were verified in AA values detected at harvest time between fruit samples un-netted and fruit samples under-netted [14].

## 5. Conclusions

Overall, the quality study of “Soreli” kiwifruit grown under the hail nets showed conflicting results.

From a specific approach, during the storage period, the SSC and firmness values of fruits under the WH nets were lower than those of fruits in the open field control sample, as was also observed for the color parameters L, b*, and Chroma (at the harvest time and from 45 days of storage).

Regarding the evolution of bioactive compounds, a decrease in chlorophylls and total carotenoids was observed starting from 30–45 days in fruits under the nets. whereas polyphenol and ascorbic acid content showed a constant trend, which was similar to the values of uncovered control from 45–60 days onwards. The flavonoids content was higher in control fruits throughout the storage time. Moreover, the fruits from vines of 8 years had significant higher values on flavonoids content compared with the fruits from vines of 9 years at harvest time.

In general, the early and late harvest time based on the °Brix values and the different ages of the kiwifruit vines under the nets did not affect the quality aspects studied in our research.

The results therefore seem to assume that the SSC and technological parameters are more affected by the reduction of natural direct radiation on the fruit (quality of the light); meanwhile, the phytochemical features seem to be more affected by the protective and shading actions of the nets against the physical and biological stress response of the kiwifruit vines.

## Figures and Tables

**Table 1:** Experimental “Soreli” kiwifruit orchards located in open field or under white nets and harvested at two different °Brix.

Orchard 1 (OF)	kiwifruit vines in open field harvested at 7–8 °Brix (8 years)
**Orchard 2**	kiwifruit vines under white net harvested at 7–8 °Brix (8 years)
**Orchard 3**	kiwifruit vines under white net harvested at 8–9 °Brix (8 years)
**Orchard 4**	kiwifruit vines under white net harvested at 7–8 °Brix (9 years)
**Orchard 5**	kiwifruit vines under white net harvested at 8–9 °Brix (9 years)

**Table 2:** Physio-chemical parameters in fruits harvested in open fields and under the white nets at harvest (0 days) and after 15 days, 30 days, 45 days, 60 days, 75 days, and 90 days of cold storage at 1 °C. Three technical replicates were realized for each biological sample.

Parameters	Treatments	0 d	15 d	30 d	45 d	60 d	75 d	90 d
**FFP (kg cm^−2^)**	Orchard 1 (OF)	6.98 ± 0.26 (dC)	4.57 ± 0.30 (cCD)	3.21 ± 0.08 (bB)	2.95 ± 0.07 (abB)	2.31 ± 0.03 (aB)	1.93 ± 0.02 (aB)	1.30 ± 0.02 (aA)
	Orchard 2	5.98 ± 0.12 (dB)	4.39 ± 0.30 (cBC)	3.17 ± 0.11 (bB)	2.74 ± 0.06 (abAB)	2.33 ± 0.02 (aA)	1.94 ± 0.04 (aB)	1.29 ± 0.02 (aA)
	Orchard 3	4.36 ± 0.34 (cA)	3.30 ± 0.15 (bA)	2.87 ± 0.08 (abA)	2.67 ± 0.05 (aA)	2.45 ± 0.03 (abAB)	1.72 ± 0.02 (aA)	1.43 ± 0.02 (aA)
	Orchard 4	6.33 ± 0.21 (dBC)	5.37 ± 0.33 (cD)	3.54 ± 0.18 (bC)	3.28 ± 0.24 (bC)	2.46 ± 0.03 (aAB)	1.93 ± 0.03 (aB)	1.42 ± 0.02 (aA)
	Orchard 5	5.89 ± 0.18 (dB)	4.01 ± 0.26 (cB)	3.02 ± 0.09 (bA)	2.61 ± 0.03 (bA)	2.32 ± 0.04 (abB)	1.54 ± 0.01 (aA)	1.35 ± 0.03 (aA)
**SSC (°Brix)**	Orchard 1 (OF)	7.53 ± 0.27 (aAB)	11.33 ± 0.30 (bBC)	13.72 ± 0.31 (cD)	14.84 ± 0.25 (cdD)	15.2 ± 0.27 (dC)	16.78 ± 0.26 (eC)	17.32 ± 0.20 (eD)
	Orchard 2	7.02 ± 0.12 (aA)	10.46 ± 0.21 (bB)	12.66 ± 0.15 (cBC)	13.15 ± 0.18 (cdC)	13.1 ± 0.18 (cA)	13.87 ± 0.13 (eB)	13.84 ± 0.14 (deB)
	Orchard 3	8.66 ± 0.09 (aB)	13.24 ± 0.26 (bD)	13.67 ± 0.30 (bcCD)	13.22 ± 0.26 (bC)	14.2 ± 023 (bcdB)	14.27 ± 0.15 (cdB)	14.7 ± 0.22 (dC)
	Orchard 4	7.07 ± 0.18 (aA)	8.43 ± 0.23 (bA)	9.99 ± 0.24 (cA)	10 ± 0.17 (cA)	14.2 ± 0.19 (eBC)	13.9 0.11 (eB)	13.9 ± 0.23 (eBC)
	Orchard 5	8.4 ± 0.19 (aB)	11.89 ± 0.27 (bC)	12.18 ± 0.27 (bB)	12.06 ± 0.40 (bB)	13.9 ± 0.27 (cAB)	12.98 ± 0.31 (bcA)	13 ± 0.24 (bcA)
**TA (mg citric**	Orchard 1 (OF)	1.52 ± 0.06 (aA)	1.45 ± 0.15 (aA)	1.11 ± 0.06 (aA)	1.46 ± 0.19 (aA)	1.38 ± 0.007 (aB)	1.66 ± 0.1 (aA)	1.51 ± 0.06 (aB)
**acid/100g FW)**	Orchard 2	1.28 ± 0.01 (bcAB)	1.32 ± 0.05 (bA)	0.96 ± 0.05 (aA)	1.23 ± 0.06 (abA)	1.19 ± 0.042 (abcAB)	1.13 ± 0.04 (abA)	1.03 ± 0.02 (abA)
	Orchard 3	0.98 ± 0.001 (aA)	1.02 ± 0.06 (aA)	1.13 ± 0.009 (aA)	1.16 ± 0.14 (aA)	1.01 ± 0.005 (aA)	1.16 ± 0.11 (aA)	1.07 ± 0.08 (aAB)
	Orchard 4	1.30 ± 0.14 (abAB)	1.37 ± 0.05 (aA)	1.04 ± 0.03 (aA)	1.38 ± 0.02 (aA)	1.42 ± 0.03 (abB)	1.66 ± 0.17 (aA)	1.50 ± 0.13 (abB)
	Orchard 5	1.37 ± 0.10 (aAB)	1.05 ± 0.09 (aA)	1.10 ± 0.02 (aA)	1.38 ± 0.09 (aA)	1.16 ± 0.11 (aAB)	1.04 ±0.07 (aA)	1.01 ± 0.04 (aA)

Capital letters reflect comparisons between different maturity stages of fruit of different orchards for each time. Small letters reflect comparisons between different storage times for each sample fruit of the same orchard. Means followed by the same letter in the same column or line do not differ from each other according to the Tukey test (*p* < 0.05). FFP = Fruit Firmness Penetrometer; SSC = soluble solids content; TA = titratable acidity.

**Table 3:** Color parameters in fruits harvested in open fields and under the white nets at harvest (0 days) and after 15 days, 30 days, 45 days, 60 days, 75 days, and 90 days of cold storage at 1 °C. Three technical replicates were realized for each biological replicate.

Parameters	Treatments	0 d	15 d	30 d	45 d	60 d	75 d	90 d
**L**	Orchard 1 (OF)	71 ± 0.42 (cC)	59.27 ± 0.65 (aA)	65.94 ± 0.40 (bD)	66.61 ± 0.35 (bC)	59.96 ± 0.50 (aD)	58.16 ± 0.44 (aD)	58.79 ± 0.32 (aC)
	Orchard 2	65.56 ± 0.62 (dB)	66.03 ± 0.72 (dC)	61.72 ± 0.39 (cC)	49 ± 0.69 (aA)	52.89 ± 0.45 (bBC)	52.5 ± 0.46 (bBC)	51.91 ± 0.36 (bB)
	Orchard 3	61.05 ± 1 (cA)	59.17 ± 0.53 (cA)	47.27 ± 0.76 (aA)	49.08 ± 0.62 (abA)	49.70 ± 0.50 (abA)	50.32 ± 0.46 (bA)	48.84 ± (abA)
	Orchard 4	65.15 ± 0.46 (dB)	64.40 ± 0.33 (dBC)	61.72 ± 0.97 (cC)	52.73 ± 0.62 (abB)	54.03 ± 0.44 (bC)	52.32 ± 0.46 (abC)	50.72 ± 0.36 (aB)
	Orchard 5	68.65 ± 0.31 (dC)	63. 45 ± 0.37 (cB)	50.58 ± 0.61 (aB)	54.62 ± 0.43 (bB)	51.67 ± 0.39 (aB)	50.8 ± 0.41 (aAB)	50.72 ± (aB)
a*	Orchard 1 (OF)	-0.54 ± 0.04 (aB)	-0.57 ± 0.05 (aB)	-0.48 ±0.03 (aC)	-0.53 ± 0.04 (aD)	-0.57 ± 0.04 (aA)	-0.60 ± 0.04 (aA)	-0.62 ± 0.04 (aB)
	Orchard 2	-0.76 ± 0.06 (abB)	-0.58± 0.05 (bB)	-0.82 ± 0.06 (abB)	-0.89 ± 0.08 (aB)	-0.65 ± 0.04 (abA)	-0.65 ± 0.04(abA)	-0.62 ± 0.05 (bB)
	Orchard 3	-0.76 ± 0.08 (bB)	-0.5± 0.04 (aA)	-1.08 ± 0.08 (aA)	-0.81 ± 0.06 (abBC)	-0.73 ± 0.05 (bA)	-0.62 ± 0.04 (bA)	-0.76 ± 0.05 (bB)
	Orchard 4	-1.85 ± 0.13 (abA)	-0.9± 0.07 (aA)	-0.71 ± 0.06 (dBC)	-0.86 ± 0.06 (abcA)	-0.61 ± 0.04 (dA)	-0.64 ± 0.03 (bcA)	-1.23 ± 0.09 (cA)
	Orchard 5	-0.73 ± 0.05 (aB)	-0.65± 0.05 (aB)	-0.7 ± 0.05 (aBC)	-0.58 ± 0.04 (aCD)	-0.67 ± 0.04 (aA)	-0.63 ± 0.04 (aA)	-0.7 ± 0.04 (aB)
b*	Orchard 1 (OF)	26.64 ± 0.29 (cdD)	21.9 ± 0.39 (aA)	27.04 ± 0.28 (cdC)	29.11 ±0.26 (eC)	26.06 ± 0.33 (cdC)	24.3 ± 0.36 (bC)	27.82 ± 0.35 (deC)
	Orchard 2	21.18 ± 0.39 (bA)	20.81 ± 0.41 (bA)	25.08 ± 0.32 (cB)	18.04 ± 0.41 (aA)	21 ± 0.39 (bA)	21 ± 0.34 (bA)	21.82 ± 0.35 (bB)
	Orchard 3	21.74 ± 0.39 (cAB)	25.21 ± 0.39 (dB)	18.08 ± 0.54 (aA)	19.37 ± 0.46 (abA)	21.43 ± 0.36 (cA)	21.32 ± 0.27 (cAB)	20.27 ± 0.36 (bcA)
	Orchard 4	22.93 ± 0.40 (bB)	26.9 ± 0.34 (cC)	26.26 ± 0.54 (cBC)	19.01 ± 0.40 (aA)	22.14 ± 0.29 (bAB)	22.14 ± 0.37 (bAB)	21.33 ± 0.32 (bAB)
	Orchard 5	25.20 ± 0.33 (dC)	26.79 ± 0.32 (eC)	19.19 ± 0.43 (aA)	23.27 ± 0.49 (cB)	23.04 ±0.33(cB)	22.4 ± 0.30 (cB)	20.82 ± 0.31 (bAB)
**Chroma**	Orchard 1 (OF)	26.65 ± 0.29 (cdD)	21.94 ± 0.39 (aA)	27.04 ± 0.28 (cdC)	29.12 ± 0.26 (eC)	26.07 ± 0.33 (cC)	24.36 ± 0.36 (bC)	27.83 ± 0.35 (deC)
	Orchard 2	21.2 ± 0.39 (bA)	20.82 ± 0.40 (bA)	25.1 ± 0.32 (cB)	18.08 ± 0.41 (aA)	21.01 ± 0.39 (bA)	21.01 ± 0.30 (bA)	21.95 ± 0.36 (bcA)
	Orchard 3	21.78 ± 0.38 (cAB)	25.23 ± 0.39 (dB)	18.15 ± 0.53 (aA)	19.4 ± (0.46 (abA)	21.45 ± 0.36 (cA)	21.33 ± 0.27 (cAB)	20.3 ± 0.36 (bcA)
	Orchard 4	23.04 ± 0.40 (cB)	27.1 ± 0.34 (dC)	26.31 ± 0.51 (dBC)	19.1 ± 0.40 (aA)	22.16 ± 0.29 (bcAB)	22.15 ± 0.37 (bcAB)	21.38 ± 0.32 (bAB)
	Orchard 5	25.22 ± 0.33 (dC)	26.8 ± 0.32 (eC)	19.22 ± 0.49 (aA)	23.28 ± 0.43 (cB)	23.05 ± 0.33 (cB)	22.46 ± 0.30 (cB)	20.84 ± 0.31 (bAB)

Capital letters reflect comparisons between different maturity stages of fruit of different orchards for each time. Small letters reflect comparisons between different storage times for each sample fruit of the same orchard. Means followed by the same letter in the same column or line do not differ from each other according to the Tukey test (*p* < 0.05). L* = lightness; a* = chromaticity coordinate from green to red; b* = chromaticity coordinate from blue to yellow; Chroma = color saturation.

**Table 4:** Carotenoids, chlorophyll a and b, polyphenols, flavonoids, and ascorbic acid content in fruits harvested in open fields and under the white nets at harvest (0 days) and after 15 days, 30 days, 45 days, 60 days, 75 days, and 90 days of cold storage at 1 °C. Three technical replicates were realized for each biological replicate.

Parameters	Treatments	0 d	15 d	30 d	45 d	60 d	75 d	90 d
**Cl a**	Orchard 1 (OF)	179.6 ± 19.9 (bA)	61.1 ± 2.78 (aA)	58.51 ± 15.1 (aAB)	48.97 ± 9.85 (aAB)	68.87 ± 9.51 (aB)	43.76 ± 1 (aC)	43.69 ± 1.15 (aC)
	Orchard 2	167.18 ± 18.06 (cA)	73.01 ± 2.53 (bA)	44.82 ± 18.74 (abAB)	47.64 ± 0.40 (abAB)	11.91 ± 0.93 (aA)	4.86 ± 2.02 (aA)	16.06 ± 1.47 (aB)
	Orchard 3	149.59 ± 18.94 (cA)	60.6 ± 14.24 (bA)	60.44 ± 0.84 (bAB)	17.74 ± 4.34 (abA)	11.02 ± 2.14 (aA)	11.73 ± 1.98 (aA)	9.36 ± 1.07 (aA)
	Orchard 4	172.82 ± 9.74 (bA)	78.22 ± 12.67 (aA)	78.08 ± 7.9 (aB)	69.32 ± 16.65 (aB)	10.85 ± 1.48 (aA)	7.80 ± 0.89 (aA)	3.80 ± 0.74 (aA)
	Orchard 5	122.39 ± 1.7 (eA)	42.95 ± 2.91 (dA)	29.56 ± 1.56 (cA)	9.46 ± 1.86 (aA)	17.89 ± 0.55 (abA)	16.30 ± 3.91 (bcB)	19.11 ± 0.82 (abB)
**Cl b**	Orchard 1 (OF)	260.15 ± 8.43 (bA)	121.49 ± 0.3 (aAB)	100.53 ± 32.87 (aA)	87.01 ± 17.7 (aB)	88.99 ± 38.45 (aB)	86.54 ± 1.54 (aC)	85.97 ± 0.78 (aD)
	Orchard 2	246.97 ± 6.6 (dA)	171.31 ± 8.71 (cBC)	95.72 ± 22.77 (bA)	89.48 ± 5.95 (bB)	24.88 ± 1.76 (aA)	20.15 ± 4.72 (aA)	37.52 ± 2.35 (aC)
	Orchard 3	188.36 ± 9.59 (cA)	117.05 ± 17.21 (bAB)	132.39 ± 5.76 (bA)	33.97 ± 8.14 (aA)	18.03 ± 2.9 (aA)	21.73 ± 2.52 (aA)	17.56 ± 2.19 (aB)
	Orchard 4	159.50 ± 26.58 (bA)	125.53 ± 13.33 (bC)	127.74 ± 13.33 (bC)	101.70 ± 10.47 (abB)	27.75 ± 4.27 (aA)	15.86 ± 2.01 (aA)	8.06 ± 1.5 (aA)
	Orchard 5	233.57 ± 1.04 (cA)	74.19 ± 14.67 (bA)	57.44 ± 2.52 (abA)	23.17 ± 1.63 (aA)	40.50 ± 4.12 (abAB)	34.20 ± 6.93 (abB)	44.43 ± 1.2 (abC)
**CAR**	Orchard 1 (OF)	39.04 ± 4.82 (cB)	18.05 ± 0.85 (bB)	20.78 ± 3.29 (bB)	19.32 ± 1.29 (bB)	11.36 ± 0.54 (aC)	7.21 ± 0.14 (aB)	7.14 ± 0.17 (aC)
	Orchard 2	35.72 ± 2.81(cAB)	17.47 ± 1.85 (bB)	16.8 ± 4.34 (bB)	17.12 ± 0.45 (bB)	4.79 ± 0.15 (aAB)	4.01 ± 0.23 (aA)	4.94 ± 0.37 (aB)
	Orchard 3	33.08 ± 0.14 (dAB)	11.44 ± 0.69 (bA)	14.41 ± 0.03 (cAB)	15.67 ± 0.16 (cB)	3.33 ± 0.25 (aA)	4.01 ± 0.23 (cB)	2.31 ± 0.23 (aA)
	Orchard 4	35.76 ± 4.64 (cAB)	18.28 ± 0.91 (bB)	23.42 ± 0.34 (bB)	23 ± 2.33 (bB)	3.9 ± 0.47 (aA)	2.72 ± 0.12 (aA)	2.05 ± 0.11 (aA)
	Orchard 5	17.75 ± 3.28 (bA)	8.61 ± 0.61 (aA)	5.57 ± 1.18 (aA)	7.21 ± 2.24 (aA)	6.16 ± 0.18 (aB)	5.33 ± 0.57 (aB)	2.42 ± 0.05 (aA)

Capital letters reflect comparisons between different maturity stages of fruit of different orchards for each time. Small letters reflect comparisons between different storage times for each sample fruit of the same orchard. Means followed by the same letter in the same column or line do not differ from each other according to the Tukey test (*p* < 0.05). Cl *a* = chlorophyll *a*; CL *b* = chlorophyll *b*; CAR = carotenoids.

**Table 5:** Polyphenols, flavonoids, and ascorbic acid content in fruits harvested in open fields and under the white nets at harvest (0 days) and after 15 days, 30 days, 45 days, 60 days, 75 days, and 90 days of cold storage at 1 °C. Three technical replicates were realized for each biological replicate.

	Treatments	0 d	15 d	30 d	45 d	60 d	75 d	90 d
**POL**	Orchard 1 (OF)	110 ± 15 (bcD)	151.2 ± 3.56 (dD)	178.22 ± 2 (eE)	188.21 ± 1.44 (eD)	142.43 ± 3.68 (cdC)	125.14 ± 2.9 (abB)	91.56 ± 1.18 (aB)
	Orchard 2	115.13 ± 2.5 (bC)	122.96 ± 1.05 (bB)	124.07 ± 3.22 (bC)	125.3 ± 0.86 (bB)	88.27 ± 6.33 (aA)	91.56 ± 3.16 (aA)	80.06 ± 3.11 (aA)
	Orchard 3	95.57 ± 3.12 (aB)	99.82 ± 3.77 (aA)	107.20 ± 3.39 (abB)	117.31 ± 3.03 (bB)	122.18 ± 0.58 (bcB)	133.91 ± 3.37 (bcB)	121.22 ± 2.49 (cB)
	Orchard 4	78.51 ± 0.53 (aA)	123.21 ± 2.94 (cB)	138.72 ± 1.42 (deD)	164.65 ± 2.04 (fC)	110.53 ± 2.18 (bB)	133.87 ± 2.29 (cdB)	147.30 ± 4.59 (efC)
	Orchard 5	87.01 ± 2.58 (abcAB)	100.53 ± 1.84 (dA)	79.44 ± 1.35 (aA)	82.01 ± 1.88 (abA)	90.48 ± 2.49 (bcdA)	94.40 ± 2.97 (cdA)	92.95 ± 1.93 (cdA)
**FLAV**	Orchard 1 (OF)	23.76 ± 0.81 (bB)	19.05 ± 0.5 (aC)	17.88 ± 0.64 (aC)	22.76 ± 0.56 (bC)	22.89 ± 0.45 (bD)	19.65 ± 0.35 (aB)	19.79 ± 0.25 (aD)
	Orchard 2	23.88 ± 0.83 (dB)	14.37 ± 0.49 (bB)	15.33 ± 0.42 (bcBC)	17.70 ± 0.7 (cB)	14.50 ± 0.26 (bC)	10.24 ± 0.22 (aA)	13.15 ± 0.32 (bC)
	Orchard 3	23.61 ± 0.51 (eB)	11.36 ± 0.66 (cA)	14.11 ± 0.54 (dB)	15.42 ± 0.55 (dB)	7.42 ± 0.04 (abA)	8.63 ± 0.52 (bA)	5.77 ± 0.51 (aA)
	Orchard 4	16.23 ± 0.81 (dA)	11.83 ± 0.49 (bcA)	13.18 ± 0.82 (cB)	17.69 ± 0.53 (dB)	6.23 ± 0.27 (aA)	9.3 ± 0.27 (bA)	9.21 ± 0.26 (bB)
	Orchard 5	12.86 ± 0.54 (bA)	11.99 ± 0.2 (bA)	9.72 ± 0.42 (aA)	10.03 ± 0.11 (aA)	11.48 ± 0.23 (abB)	10.12 ± 0.48 (aA)	9.68 ± 0.44 (aB)
**AA**	Orchard 1 (OF)	70.40 ± 0.7 (dC)	66.58 ± 1.65 (bcdC)	68.01 ± 1.87 (dB)	65.56 ± 2.87 (cdC)	58.44 ± 3.56 (abcB)	57.32 ± 2.08 (abC)	53.34 ± 1.72 (aC)
	Orchard 2	58.9 ± 2.52 (cB)	46.01 ± 3.03 (abAB)	43.14 ± 2.44 (aA)	48.73 ± 1.19 (abcAB)	53.53 ± 1.57 (bcAB)	54.26 ± 2.24 (bcBC)	51.72 ± 0.94 (abcBC)
	Orchard 3	38.03 ± 1.77 (aA)	43.79 ± 2.51 (aA)	42.80 ± 1.48 (aA)	41.09 ± 1.89 (aA)	45.71 ± 3.9 (aAB)	46.44 ± 1.03 (aAB)	44.74 ± 1.92 (aAB)
	Orchard 4	45.24 ± 3.17 (aA)	53.26 ± 0.6 (aB)	48 ± 3.91 (aA)	52.16 ± 2.58 (aB)	43.76 ± 2.25 (aA)	44.79 ± 0.7 (aA)	41.93 ± 1.81 (aA)
	Orchard 5	42.28 ± 2.87	47.49 ± 1.37 (abAB)	51.23 ± 2.64 (abA)	55.88 ± 2.45 (bB)	48.57 ± 2.15 (abAB)	55.67 ± 1.77 (bC)	53.42 ± 1.20 (bC)

Capital letters reflect comparisons between different maturity stages of fruit of different orchards for each time. Small letters reflect comparisons between different storage times for each sample fruit of the same orchard. Means followed by the same letter in the same column or line do not differ from each other according to the Tukey test (*p* < 0.05). POL = polyphenols; FLAV = flavonoids; AA = ascorbic acid.

## Data Availability

Data is contained within the article.
